# A Compassion-Focused Online Single-Session Intervention to Reduce Academic Stress in University Students: Protocol for a Randomized Controlled Trial

**DOI:** 10.2196/83850

**Published:** 2026-05-22

**Authors:** Clara Wehlage, Sofija Zivanovic, Helen Koechlin, Nathalie Sarah Schicktanz, Olivia Bolt, Dorothée Bentz

**Affiliations:** 1 Clinical Psychology and Translational Psychotherapy Research, Faculty of Psychology, University of Basel, Missionsstrasse 62A, Basel, Basel-City, 4055, Switzerland, 41 612070229; 2 Department of Psychosomatics and Psychiatry, University Children’s Hospital, University of Zurich, Zurich, Switzerland; 3 Children’s Research Centre, University Children’s Hospital Zurich, University of Zurich, Zurich, Switzerland; 4 Department of Psychology, Division of Child and Adolescent Health Psychology, University of Zurich, Zurich, Switzerland; 5 Cognitive Neuroscience, Department of Biomedicine, University of Basel, Basel, Basel-City, Switzerland

**Keywords:** academic stress, university students, single-session intervention, compassion-focused therapy, internet-delivered

## Abstract

**Background:**

Among university students, the experience of academic stress is associated with symptoms of anxiety and depression. Single-session interventions (SSIs) are structured and accessible self-guided interventions, often delivered digitally, that integrate elements of empirically supported treatments. Compassion-Focused Therapy (CFT) has been shown to be beneficial in alleviating stress, anxiety, and depression. However, the effectiveness of CFT (1) in the academic context, and (2) when delivered in brief digital formats, has not yet been tested.

**Objective:**

This study aims to evaluate the effectiveness of a compassion-focused online SSI for reducing stress among university students.

**Methods:**

University students aged 18‐65 years that indicate at least mild stress (a score >7 on the stress scale of the Depression Anxiety Stress Scale–21 [DASS-21]) will be recruited from German-speaking Switzerland. The target sample size is 156 participants randomly assigned to either the intervention or the waitlist control group. The 45-minute intervention follows the typical SSI structure and combines psychoeducation on academic stress, core CFT principles, guided breathing, compassion imagery exercises, and includes an action plan. Assessments will be conducted at baseline, 24 hours after the intervention, and at the 1-week follow-up. The primary outcome is the change in stress between baseline and 1-week follow-up, measured with the stress scale of the DASS-21. Secondary outcomes include depressive symptoms and anxiety measured with the depression and anxiety scales of the DASS-21 between baseline and 1-week follow-up. Data will be analyzed using linear (mixed-effects) models.

**Results:**

Ethical approval for the study was received in September 2025. Recruitment and data collection started in November 2025; data collection will conclude once 172 participants have been enrolled. As of April 15, 2026, 67 participants have been enrolled. Data analysis is planned for winter 2026, with the primary findings being prepared for publication in the spring of 2027.

**Conclusions:**

To our knowledge, this study is the first randomized controlled trial to examine the effects of a compassion-focused online SSI on reducing stress in university students. If the program proves to be effective, it would offer a low-threshold, cost-effective, and scalable mental health intervention for the academic context.

## Introduction

### Academic Stress in University Students

According to Lazarus and Folkman [1], stress arises when individuals perceive a discrepancy between the demands of a situation and their available resources. In the academic context, students often experience stress when the perceived demands of heavy workloads, tight deadlines, and high-performance expectations exceed their available coping resources [2,3]. Psychological contributors to academic stress include fear of failure, perfectionism, and self-criticism [4]. These are associated with maladaptive coping responses such as avoidance, overcompensation, or emotional suppression [4,5].

Recent studies from Germany report high levels of strain among university students, with more than 30% experiencing frequent depressive symptoms, more than 40% having difficulties with concentration and decision-making, and around 40% to 50% feeling exhausted or showing signs of burnout [6,7]. The proportion of students who perceive negative effects of academic stress on their health varies by gender, with almost 40% of students who identify as female, two-thirds of students as gender-diverse, and 28% of students as male reporting affected health [6,8]. In the Netherlands, a survey of 224,000 university students found that 39.8% experienced high stress related to their academic workload, 68% felt psychologically burdened by study demands, and 35% reported frequent loneliness, with 6.9% reporting the experience to be severe [9]. Similar patterns were found in Switzerland, where a cross-sectional study among 915 university students revealed that lower academic satisfaction was strongly associated with higher levels of perceived stress, symptoms of anxiety, and depression [10]. These high prevalence rates of psychological distress coincides with persistently underresourced student support services [11,12], reflecting a significant treatment gap for university populations [13].

### Interventions to Counter Academic Stress

Meta-analytic evidence shows that cognitive, behavioral, and mindfulness-based interventions effectively reduce anxiety, depression, and physiological stress responses among university students [14]. Recent findings highlight the benefits of mindfulness, acceptance, and self-compassion components in improving emotional regulation and reducing self-critical thought patterns [15]. However, few approaches explicitly target fear of failure and self-criticism, key psychological drivers of academic stress. Compassion-Focused Therapy (CFT) directly addresses these mechanisms by cultivating self-compassion and balancing affiliative and threat-based emotional systems.

### CFT

CFT is an evolution-informed, biopsychosocial therapy model, which was developed by Gilbert [16]. It aims to cultivate compassion in order to alleviate psychological distress. Based on research into the neurophysiology of emotions, at least three types of emotion regulation systems can be distinguished: a threat and protection system, a drive and excitement system, and a soothing and social safeness system [16]. CFT works on addressing imbalances between these three systems. Empirical evidence shows that CFT can reduce negative mental health outcomes such as depression and self-critical thinking, while promoting greater self-compassion and compassion toward others [17-19].

In the context of academic stress, higher self-compassion has been associated with reduced academic stress and improved psychological well-being among students [20]. Similarly, Kotera et al [21] found that self-compassion was a significant predictor of better mental health (ie, decrease in symptoms of anxiety and depression) among UK social work students. Compassion-based interventions have also been shown to enhance resilience and reduce distress in student nurses [22], as well as to improve self-criticism, compassion, depression, anxiety, and shame in Chinese international students in South Korea [23]. These findings provide empirical evidence for applying compassion-focused interventions to student populations experiencing academic stress.

### Single-Session Interventions (SSIs)

As the university setting is characterized by high demands and limited resources, brief, scalable, and accessible programs appear particularly suitable. Online single-session interventions (SSIs) are brief, structured psychological programs delivered in one sitting or during one contact with an online program. They are designed to provide immediate support or skills training for specific mental health concerns [24] and have shown positive effects in multiple mental health contexts. For example, Schleider et al [25] found that a single online SSI reduced depressive symptoms and feelings of hopelessness in adolescents, while increasing their sense of agency. The effects were observed for up to three months. Further, a meta-analysis found SSIs to be associated with significant beneficial effects across psychiatric problems in youth [26]. Although SSIs have generally been shown to improve mental health outcomes [25], their application to stress reduction in higher-education settings remains unexplored.

### Research Gap

Despite the high prevalence of psychological distress among university students [8], randomized controlled trials testing low-threshold, accessible, and scalable interventions specifically targeting academic stress remain rare. Most existing interventions are relatively time-intensive, typically involving multiple sessions over several weeks [14,15]. The potential of compassion-focused approaches in this context is largely untapped, especially when they are delivered digitally in a single session. Addressing this gap is critical for developing evidence-based, accessible, cost-effective strategies to support mental health in student populations.

### Research Objectives and Hypotheses

The primary objective is to evaluate the effectiveness of a compassion-focused online SSI in reducing self-reported stress among university students. The secondary objective is to evaluate the effectiveness of the SSI in reducing depressive symptoms and anxiety. The study will test four hypotheses: (1) The intervention group will show a greater reduction in perceived self-reported stress (DASS-21 stress scale) from baseline to 1-week follow-up than the waitlist control group, (2) the intervention group will show a greater reduction in depressive symptoms (DASS-21 depression scale) than the waitlist control group from baseline to 1-week follow-up, (3) the intervention group will show a greater reduction in symptoms of anxiety (DASS-21 anxiety scale) than the waitlist control group from baseline to 1-week follow-up. Furthermore, we expect that (4) the intervention group will report higher improvements in self-compassion from baseline to postintervention and 1-week follow-up compared to the waitlist control group.

## Methods

### Trial Design

**Figure 1.**
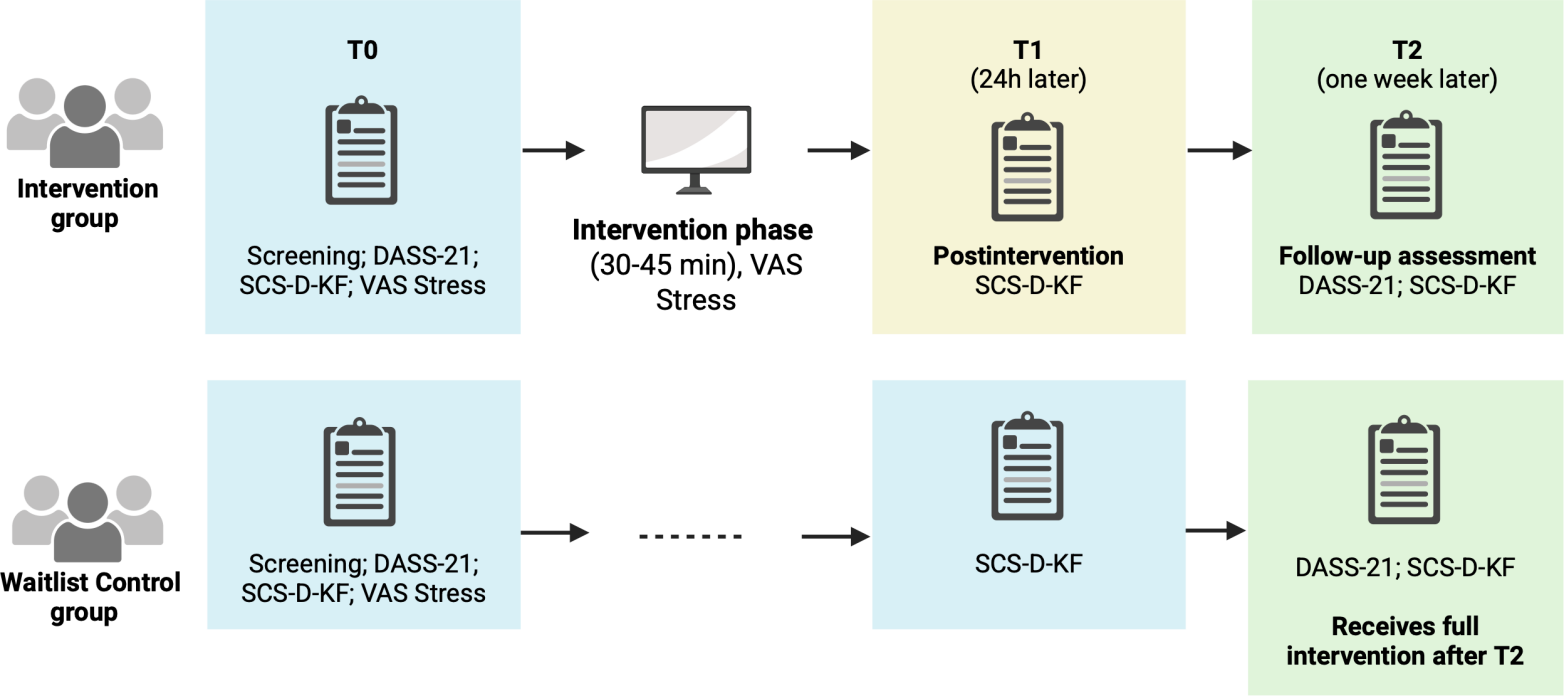
Overview of the trial. DASS-21: Depression, Anxiety, and Stress Scale - 21 items, SCS-D-KF: German 12-Item Short Form of the Self-Compassion Scale, VAS Stress: Visual Analogue Scale Stress.

This design is a randomized, controlled, parallel-group trial with 2 arms (intervention vs waitlist control) (Figure 1). No additional interventions will be provided beyond the intervention itself during the research period. Assessments will be carried out at baseline (T0), 24 hours after the intervention (T1), and at 1-week follow-up (T2). The trial protocol has been registered at the Open Science Framework [27] and the German Clinical Trial Register (Identifying number DRKS00038409), including prespecified outcomes and a statistical analysis plan (Figure 1).

### Ethical Considerations

The research plan version 1 (August 31, 2025) and informed consent form were approved by the Ethics Committee of the Faculty of Psychology at the University of Basel, Switzerland, with the ethics case number 029-25-1 on September 15, 2025. On October 30, 2025, an amendment to research plan version 1 (October 23, 2025), introducing an additional inclusion criterion to ensure that participants present with a measurable level of stress at baseline, was approved by the responsible ethics committee (ethics case number 029-25-2) prior to study commencement. Any further protocol amendments will be submitted to the responsible ethics committee for approval and will be transparently communicated. The principal investigators (DB and OB) are responsible for updating the trial registration in the German Clinical Trials Register and ensuring that all relevant parties are informed, as required.

To ensure confidentiality and data security, all study-related data will be stored on a restricted-access server. The server is managed by the Faculty of Psychology’s IT department. Data will be pseudoanonymized by assigning a unique study ID upon enrollment; no personally identifiable information will be stored permanently. The online questionnaires will have built-in checks to ensure all items are completed before submission, minimizing missing data.

During the informed consent, participants will be informed that the two principal investigators (DB and OB), both licensed psychotherapists, may be contacted at any time should questions arise or if psychological discomfort related to participation exceeds a level that they feel comfortable handling independently. Additionally, before each exercise of the intervention, a safety note advises that focusing on internal sensations may temporarily increase discomfort for some individuals and recommends pausing the practice if deemed necessary. Given the minimal-risk, single-session, automated, and self-guided nature of the intervention, adverse events will not be systematically assessed. Participants will be informed that they may discontinue participation at any time and may contact the principal investigators if they experience any concerns or unintended effects. If needed, participants will receive guidance on how to access appropriate psychological support.

Participants will not receive direct monetary compensation, but they will have the opportunity to enter a raffle at the end of the study. Twenty winners will be selected at random, and each will receive a book voucher worth 50 CHF (US $64.02). Participation in the raffle will be voluntary, and winners will be contacted via email once data collection is complete. The use of book vouchers as study incentives is well justified, as meta-analytic evidence suggests that material incentives increase both response and retention rates in web-based studies [28,29].

### Procedure

Eligible students will be invited to participate via a website specific to the study, where they will find detailed information about the study procedures. Participants will be asked to provide electronic informed consent before completing an anonymous screening questionnaire to assess their eligibility. The questionnaire is hosted online on the LimeSurvey platform [30]. Participants who meet all inclusion criteria will continue with the baseline assessment (T0) (Figure 1). Those who do not meet the inclusion criteria will be informed that they are not eligible to participate. Participants will complete the intervention and all assessments online.

### Randomization and Blinding

Prior to recruitment, a computer-generated randomization sequence will be created using R [31,30] with a 1:1 allocation to the intervention group and waitlist control group. Simple randomization without blocking or stratification will be used. Participants will be added sequentially to the pregenerated randomization sequence by the personnel responsible for enrollment. For procedural reasons, participants will be randomized before all inclusion and exclusion criteria have been fully verified, as group-specific access links must be provided immediately after successful screening. In the event of a screening failure, the next eligible participant will replace the excluded individual. Once enrolled, the intervention and all subsequent outcome assessments will be made available automatically on the study platform. Automated email reminders to fill in outcome assessments at T1 and T2 will be programmed at enrollment. Investigators do not interact with participants regarding allocation. The personnel enrolling participants have access to the randomization list solely for the purpose of sequentially adding participants. They do not have any influence over assignment beyond adding participants to the list. After a participant is added, allocation, intervention delivery, and outcome assessments are fully automated, preventing any post-enrollment influence by investigators. Participants are not explicitly informed of their group assignment, although they may infer it from the information provided during the informed consent process. As the intervention and outcome assessments are fully automated, no blinding of investigators is required. The data analyst responsible for statistical analyses will remain blinded to group allocation, with group labels coded to ensure that the data analyst is unaware of which condition corresponds to the intervention or waitlist control group during data analysis.

### Recruitment and Eligibility Criteria

The participants will be university students from German-speaking universities in Switzerland. The trial will be advertised on German-speaking universities’ campuses through flyers and brief announcements in teaching formats, as well as institutional newsletters and social media channels of the participating universities. In addition, targeted social media advertisements will be used. Interested individuals will be directed to a study-specific website hosted by the Swiss provider Infomaniak [32], where they will receive detailed information on how to enter the trial.

To be eligible for participation, individuals (1) must be healthy students enrolled at universities in German-speaking Switzerland, (2) be between 18 and 65 years of age, (3) be fluent in German, (4) not be currently undergoing psychological or psychiatric treatment, (5) have a score >7 on the DASS-21 stress scale, and (6) be willing to participate in the study. Participants will be excluded if they (1) are currently in psychological or psychiatric treatment, (2) have insufficient German language skills to understand study instructions, or (3) score ≥10 on the DASS-21 depression scale and/or ≥2 on item 21 (“I felt that life was meaningless“) during screening. These data will be collected through self-report questionnaires, including questions such as: “Are you currently undergoing psychotherapy?” to check for inclusion criteria (4).

### Rationale and Development of the Intervention

The compassion-focused online SSI was developed specifically for this study to address academic stress among university students. Grounded in the theoretical framework of CFT [19,33], the intervention draws upon evidence-based psychoeducational and experiential techniques. The content was developed based on previous research into stress management in higher education settings and by established CFT protocols. The study team created and recorded all materials, including video content, guided audio exercises, and written instructions, with support from the New Media Center of the University of Basel. Two members (CW and SZ) of the study team were Master’s students, and additional Master’s students pilot-tested the intervention, providing feedback that was used to adapt and further refine the content. Materials were then pilot-tested internally to ensure clarity, accessibility, and engagement .

### Delivery Platform

The intervention is delivered online via a study-specific website hosted by the Swiss provider Infomaniak. Participants receive a personal access code via email, granting them entry to the intervention platform. All content is self-guided and designed to be completed in a single 45-minute session, with no involvement from a live facilitator. The process is fully automated, enabling participants to proceed at their own pace while following the structured sequence of components.

### Components of the SSI

#### Intervention Structure

**Figure 2.**
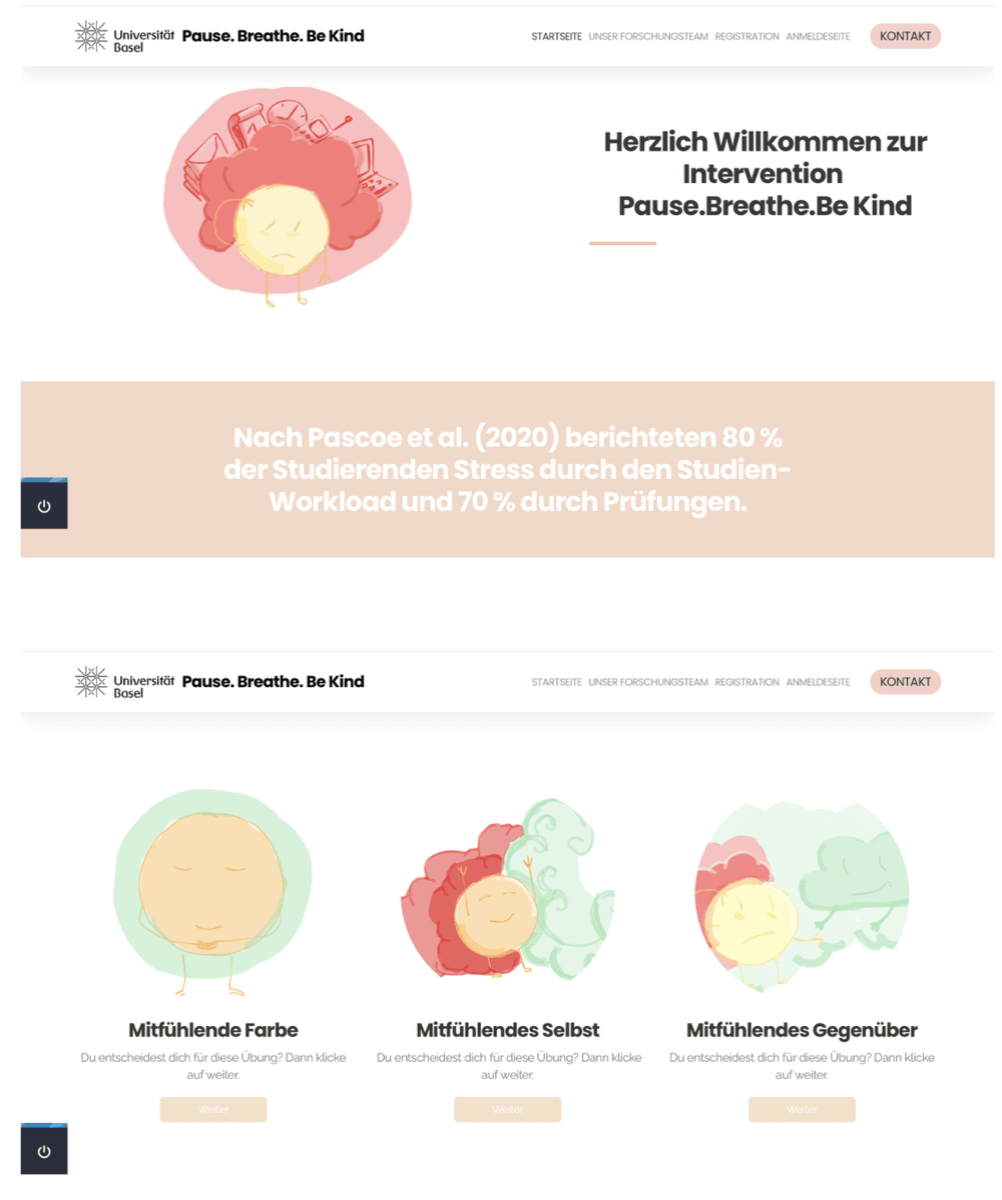
Screenshots of the intervention.

The intervention comprises 3 sequential modules consisting of a psychoeducational component, an experiential component, and a final reflection and action planning phase (Figure 2 for an overview of the intervention), in accordance with the structure of other SSIs [24,34-36].

#### Psychoeducational Component

The session begins with a 3-minute animated explainer video [37] that introduces the concept of academic stress and its psychological impact. The video draws on current research on stress and student well-being [38]. Participants are then invited to reflect briefly on their own experiences of academic stress, a process expected to take them 3 minutes (see supplementary material, available on the Open Science Framework [39]).

Participants are then introduced to the core principles of CFT with a prerecorded 6-minute video from the author OB, who is a CFT specialist and licensed psychotherapist. In the video, the 3-circle model of emotion regulation (threat, drive, and soothing systems) and the role of compassion in managing self-criticism and distress are explained, combining theoretical background from evolutionary psychology, neuroscience, and clinical research. Next, participants are asked to spend about 3 minutes reflecting on their personal experiences of compassion.

#### Experiential Component

In the experiential component, participants complete a guided 5-minute soothing rhythm breathing exercise, during which they adopt and practice a compassionate body posture and a calm breathing rhythm. This exercise is followed by 1 of 3 compassion imagery exercises (compassionate color, compassionate self, or compassionate friend) to practice self-compassion skills, which will take approximately 6 minutes. All exercises will be provided to the participants in audio and written formats, enabling participants to select their preferred method (screenshots of the intervention are shown in Figure 2). Finally, participants are asked to reflect for about 3 minutes on the intervention session in its entirety.

#### Reflection and Action Planning

After the experiential activities, participants receive a PDF “Action Plan” summarizing key psychoeducational points and specific strategies for applying self-compassion in situations of academic stress. The Action Plan also contains information on further mental health resources. The corresponding materials will be uploaded upon publication of the study on the Open Science Framework [39]. Participants can access all intervention components at any time after completion using their personal access code.

### Control Group

Participants allocated to the waitlist control group will not receive the intervention until they have completed all the study assessments (T0, T1, and T2). They complete the same questionnaires as the intervention group at the same time points, enabling direct comparisons between the 2 groups.

### Adherence, Retention, and Discontinuation

Access to the intervention will be provided to participants in the intervention group directly after enrollment in the trial. Participants in the waitlist control group will receive access to the intervention after the completion of the final follow-up assessment (T2). No additional measures will be implemented to enhance adherence due to the single-session, automated, and self-guided nature of the SSI, and no ongoing investigator involvement will be required.

Several strategies will be used to promote participant retention. First, the overall time burden of the intervention and outcome assessments is less than an hour. Second, the follow-up intervals are short, with assessments conducted 24 hours and 7 days after the intervention. Third, participants will receive automated reminders to complete the outcome assessments at each follow-up assessment. Finally, participants will be offered the opportunity to enter a raffle as compensation for their participation.

Participants may withdraw from the trial at any time without consequences. No further data will be collected from participants after withdrawal. No investigator-initiated withdrawal will occur unless participants actively request withdrawal. Participants who discontinue the intervention or request withdrawal will be classified as dropouts, as the intervention cannot be adapted, modified, or resumed once discontinued. Loss to follow-up at each assessment time point, including T2, will be recorded.

### Outcomes

All outcomes will be collected online. Basic demographic data (age, gender, and academic level) will be collected at baseline (T0). Change in perceived stress, the primary outcome, will be assessed using the difference score of the stress scale of the German version of the DASS-21 [40], measured at baseline (T0) and at 1-week follow-up (T2). The stress scale of the DASS-21 consists of 7 items rated on a 4-point Likert scale (0 = “did not apply to me at all” to 3 = “applied to me very much or most of the time”), yielding total scores from 0 to 21 for the difference score. Higher difference scores indicate greater reduction in perceived stress.

The secondary outcomes are the change in depressive symptoms and anxiety. Change in depressive symptoms and anxiety will be assessed using the difference score of the respective DASS-21 scales, measured at T0 and T2. Each scale consists of seven items, which are rated on the same 4-point Likert scale as the stress scale (total difference score range: 0‐21). The additional exploratory outcome, self-compassion, will be measured using the short version of the German Self-Compassion Scale (SCS-D-KF) [41]. This scale comprises 12 items covering 6 dimensions of self-compassion. Each item is rated on a 5-point Likert scale (1=“almost never” to 5=“almost always”). The scale will be administered at T0, T1, and T2. The primary predictor variable is group allocation (intervention vs. waitlist control), which is assigned through randomization and coded dichotomously (0=control, 1=intervention). No data monitoring committee has been established due to the minimal-risk, single-session, fully automated nature of the intervention. Oversight will be provided by the principal investigators.

### Statistical Methods

Analyses will follow a complete-case per-protocol (CC-PP) approach, including only participants who completed T0, T1, and T2 (complete cases). Inclusion and exclusion criteria for PP are prespecified in the protocol. Linear models in combination with type II ANOVA will be applied to investigate group differences for the primary and secondary outcomes.

The primary outcome is the change in perceived stress (DASS-21 stress scale) from baseline (T0) to 1-week follow-up (T2). Secondary outcomes are (1) change in depression (DASS-21 depression scale) from T0 to T2, and (2) change in anxiety (DASS-21 anxiety scale) from T0 to T2. Group allocation (intervention vs waitlist control) will be included as a between-subject factor.

For the additional outcome self-compassion (SCS-D-KF) (3), which is assessed at T0, T1, and T2, a linear mixed-effects model will be used, including visit (T1 and T2) as a within-subject factor and group as a between-subject factor. Subject ID will be included as a random effect to account for within-subject correlations across visits. Baseline self-compassion (T0) will be included as a covariate to adjust for individual differences at study entry.

Covariates (sex, age, and academic level) will be entered as main effects and as 2-way interactions with group. Nonsignificant interaction terms will be removed from the final model. If significant interactions are detected, post hoc tests will be conducted to explore and describe the effects.

Group differences in change scores of the Visual Analogue Scale stress from T0 to directly after the intervention will be analyzed as a manipulation check to assess whether the intervention produced the expected immediate effect on perceived stress.

Results will be reported as mean (SDs) for the intervention and control groups, 2-sided *P* values, and adjusted between-group differences with 95% CIs. For the secondary outcomes, the significance level will be adjusted using a Bonferroni correction for the number of independent tests. Effect sizes will be expressed as Cohen *d*, derived from the *t* values of the models and adjusted for covariates. By convention, *d*=0.2 will be interpreted as a small effect, *d*=0.5 as a medium effect, and *d*=0.8 as a large effect.

All analyses will be performed using R (version 4.5.X; R Development Core Team) [31].

### Attrition and Assessment of Potential Bias Under the CC-PP Approach

As the primary analyses follow a CC-PP approach requiring observed data at T0, T1, and T2, potential bias due to attrition will be assessed. Completion rates will be summarized by randomized group and compared using chi-square or Fisher exact tests, as appropriate. Baseline characteristics will be compared between completers and noncompleters, prioritizing the corresponding T0 value of each primary and secondary outcome, using appropriate statistical tests and standardized mean differences.

To formally evaluate differential attrition while accounting for baseline outcome severity, dropout will be modeled separately for each outcome using logistic regression with treatment group as the primary predictor, adjusted for the respective outcome’s T0 value as well as age and sex (dropout ~ group+outcome_T0+ age+sex). If the number of dropout events is insufficient to support covariate-adjusted models, parsimonious models will be fitted as prespecified. Sensitivity analyses under an intention-to-treat framework using linear mixed-effects models with all available repeated measures will be conducted to assess the robustness of the per-protocol findings to different assumptions about missing data.

### Sample Size

We conducted an a priori power analysis for an independent-samples *t* test (2-tailed, *α*=.025, power=0.80, allocation ratio=1). The adjusted significance level reflects our decision to control for 2 independent tests corresponding to the 2 secondary hypotheses. Assuming a medium effect (Cohen *d*=0.5) for our primary outcome the change in perceived stress, the required total sample size was estimated as n=156 (78 per group; G*Power 3.1.9.6). Only participants who successfully pass screening will be counted toward the target sample size. Recruitment will be considered complete once 86 eligible participants per group have passed screening. Accordingly, 86 participants per group will be randomized. Assuming an expected attrition rate of 10% per group, we anticipate a final sample of 156 participants, with 78 participants in each group. Since SSIs are generally expected to yield effects in the small-to-medium range, with the strongest effects observed immediately after the intervention and up to 2 weeks later [26], a moderate effect size was deemed the most appropriate estimate.

## Results

**Figure 3.**
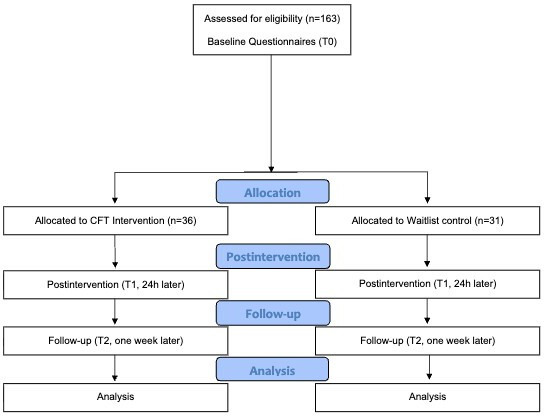
CONSORT (Consolidated Standards of Reporting Trials) flow diagram (included data as of April 15, 2026). CFT: Compassion-Focused Therapy.

As of April 15, 2026, 67 participants have been enrolled. The CONSORT (Consolidated Standards of Reporting Trials) flow diagram is shown in Figure 3. Data collection will conclude once the target sample size of 156 completed cases is reached or 172 participants have been enrolled and ended their participation, after which data analysis will begin immediately. As the study does not include clinical populations and is of minimal risk, no interim analyses or stopping guidelines are planned. Results will be published after data collection, presented at scientific conferences, posted on the German Clinical Trials Register (DRKS00038409), and made available as a preprint. The principal investigators (DB and OB) will lead the preparation of the manuscript reporting the trial results. All investigators who contribute substantially to study design, conduct, data analysis, or interpretation will be offered authorship in accordance with International Committee of Medical Journal Editors (ICMJE) guidelines.

Demographic characteristics, including age, sex, gender, and academic level, will be reported to provide a comprehensive description of the study population. The primary analysis will focus on stress reduction and follow a CC-PP approach. Subgroup analyses will be conducted to explore variations in intervention effects across relevant participant characteristics, such as baseline self-compassion or academic level.

## Discussion

### Expected Findings

This study aims to contribute to and expand upon the existing interventions to counter academic stress in university students with a new approach combining the principles of single-session interventions with CFT. One expected finding is a reduction in self-perceived stress following participation in the intervention at 1-week follow-up. Previous research has demonstrated that compassion-focused approaches can effectively reduce psychological distress, including stress, depressive symptoms, and self-criticism, while increasing self-compassion in diverse populations [18-20]. This study builds on these findings by evaluating a fully digital SSI, which provides low-barrier access for students who may face time constraints, stigma, or a lack of on-campus psychological support.

Another anticipated outcome of this study is the provision of empirical evidence on the feasibility and acceptability of a digital CFT-based SSI for university populations. By combining psychoeducation on academic stress with experiential compassion exercises, the intervention addresses potential maintenance factors of mental distress, such as self-criticism, with a combination of theoretical and practical content [4,20,22]. Examining the temporal course of the intervention’s effects will provide valuable insights into how these effects evolve over time.

Finally, the study anticipates that the intervention will be scalable and cost-effective. Due to its brief duration, minimal resource requirements, and self-guided nature, the intervention could be integrated into existing university mental health services or offered independently via online platforms. Previous studies on SSIs have highlighted their potential for widespread dissemination at low cost [24,25,42], making them particularly well-suited to contexts where institutional support services are underresourced. Therefore, the overarching aim of this study is to advance the empirical understanding of scalable, low-threshold mental health interventions that are tailored to the realities of academic life.

### Limitations

As the intervention and assessments are conducted entirely online, there is a higher risk of attrition compared with in-person formats. To address this, multiple retention strategies will be implemented, including automated reminder emails and multiple prompts for incomplete follow-up surveys. Participants will also be provided with clear, engaging instructions for the study. However, dropouts may still occur, and differential attrition could affect the statistical power and the representativeness of the final sample. To mitigate this, differential attrition will be formally assessed using logistic regression, and the robustness of the findings will be evaluated via sensitivity analyses within an intention-to-treat framework.

Another limitation concerns the generalizability of the findings. Students who are more comfortable with digital technology and online learning formats may be more likely to participate and engage fully, whereas those with limited digital literacy skills may be underrepresented. This may limit the generalizability of the findings to populations with lower technology access, preference for or experience with digital formats. These populations might require alternative formats or supplementary onboarding support. Additionally, no feedback or satisfaction ratings regarding the intervention experience are collected from the participants upon completion of the program. As a result, insights into participants’ perceptions of the intervention, potential barriers to engagement, and reasons for discontinuation remain limited. Collecting qualitative and quantitative feedback in future studies would be valuable for improving the intervention content, delivery, and recruitment strategies. Moreover, such feedback could help to better understand the underlying causes of attrition and inform targeting measures to reduce dropout rates in subsequent online interventions.

### Potential for Broader Application

Although the intervention was specifically designed to address academic stress in university settings, its underlying principles are not limited to this population. The psychoeducational and experiential components of CFT target transdiagnostic processes such as self-criticism, perfectionism, and difficulties in emotion regulation, which are relevant across a wide range of contexts. It is therefore conceivable to adapt the content to other groups experiencing similar stressors, such as high school students, health care professionals, or employees in high-pressure work environments.

### Conclusions

In conclusion, this study will evaluate the effectiveness of a compassion-focused, online SSI for alleviating academic stress among university students. Leveraging digital delivery and the brief SSI format, the intervention would offer a promising, accessible approach to mental health support in academic contexts where psychological strain is high and available services are limited. If the intervention proves to be effective, the intervention would be easily scaled up and adapted across institutions to provide a cost-effective way of promoting resilience, self-compassion, and stress reduction among a population facing significant mental health challenges. Beyond its relevance to student well-being, the findings will contribute to the growing body of evidence for SSIs and inform the development of scalable digital mental health strategies in higher education.

## Abbreviations

CC-PP: complete-case per-protocol
CFT: Compassion-Focused Therapy
CONSORT: Consolidated Standards of Reporting Trials
DASS-21: Depression, Anxiety, and Stress Scale – 21 Items
SCS-D-KF: German 12-Item Short Form of the Self-Compassion Scale
SSI: single-session intervention
